# Postpartum Eclampsia Complicated With Posterior Reversible Encephalopathy Syndrome

**DOI:** 10.7759/cureus.20799

**Published:** 2021-12-29

**Authors:** Niruby Rasendrakumar, Luxhman Gunaseelan, Sai S Muthyala, Meyyappan Meenakshisomasundaram, Nidhi Sharma

**Affiliations:** 1 Obstetrics and Gynecology, Saveetha Medical College, Chennai, IND; 2 Internal Medicine, Saba University School of Medicine, Devens, USA; 3 Radiology, Saveetha Medical College, Chennai, IND

**Keywords:** magnetic resonance imaging, vasogenic edema, reversible posterior leukoencephalopathy syndrome (rpls), postpartum eclampsia, posterior reversible encephalopathy syndrome (pres)

## Abstract

Posterior reversible encephalopathy syndrome is an uncommon yet devastating neurological maternal complication in pregnancy. Patients typically present with headache, visual disturbances, nausea, or altered mental status, and may develop seizures or loss of consciousness. Imaging usually reveals sub-cortical vasogenic edema at the parietal and occipital lobes. We discuss a case of a 29-year-old patient who developed posterior reversible encephalopathy syndrome secondary to postpartum eclampsia. The diagnosis was made after magnetic resonance imaging revealed hyperintensities in the bilateral posterior parietal, occipital and frontal lobes. The patient’s symptoms resolved after prompt treatment with levetiracetam and labetalol. Peer-reviewed publications were then sourced from online databases to explore the etiology, clinical presentation, and management of posterior reversible encephalopathy syndrome. Our results were compared with the existing data. However, the rarity of posterior reversible encephalopathy syndrome following postpartum eclampsia in the obstetric population meant limited literature existed. Therefore, the case report is novel. Combined with findings from the literature, our results from the case report supported our findings that prompt diagnosis and management are the keys to reverse posterior reversible encephalopathy syndrome.

## Introduction

Posterior reversible encephalopathy syndrome is a rare neurological condition that can occur in the obstetric population shortly after delivery [1]. Posterior reversible encephalopathy syndrome has various etiologies such as hypertensive encephalopathy, renal failure, autoimmune disorders, and sepsis [1,2]. Patients with posterior reversible encephalopathy syndrome present with nonspecific clinical signs and symptoms, such as headache, vomiting, visual disturbances, and altered mental status [2]. Magnetic resonance imaging (MRI) is typically used to confirm the diagnosis, revealing sub-cortical vasogenic edema at the bilateral parietal and occipital lobes [3]. In the obstetric population, pre-eclampsia and eclampsia are the most common causes of posterior reversible encephalopathy syndrome [4]. Pre-eclampsia is a syndrome occurring during pregnancy or in the postpartum period, characterized by hypertension and proteinuria [5]. Eclampsia is diagnosed as the presence of pre-eclampsia with seizures that cannot be explained by other causes [5]. The majority of both pre-eclampsia and eclampsia cases occur between 20 weeks of pregnancy and 48 hours postpartum, but may occur up to four weeks after delivery [6].

Most cases of eclampsia occurring during the late postpartum period are preceded by pre-eclampsia with imminent signs and symptoms. However, as symptoms of pre-eclampsia are usually not reported, only less than 22% of patients with posterior reversible encephalopathy syndrome have a previous diagnosis of pre-eclampsia [7]. As such, the opportunity to prevent the progression of pre-eclampsia to eclampsia and posterior reversible encephalopathy syndrome is lost.

This case report describes a patient who developed posterior reversible encephalopathy syndrome secondary to eclampsia, without any prior diagnosis of pre-eclampsia. A literature review was also conducted to elucidate the pathophysiology, common clinical presentation, ideal diagnostic and management protocol, and potential complications associated with posterior reversible encephalopathy syndrome.

## Case presentation

A 29-year-old South Asian female, gravida 1 para 0 at 38 week + 4 day gestation, had a spontaneous onset of labor and presented to the obstetrics unit for delivery. Her body mass index prior to conception was 31 kg/m^2^. At the time, she reported a one-day history of cramping lower abdominal pain. She was diagnosed with gestational diabetes mellitus at 28 weeks, after her glucose tolerance testing revealed a two-hour plasma glucose of 170 mg/dL and a three-hour plasma glucose of 150 mg/dL. Her gestational diabetes was well controlled on a diabetic diet. She has no other past medical history. Her family history included an older sister with a history of pre-eclampsia. She was prescribed aspirin one month prior to delivery as she was at moderate risk for pre-eclampsia [8]. She denied any headache, visual disturbances, or altered mental status. Antenatal ultrasound preformed at 34 weeks gestation were normal.

On admission, the patient was found to be afebrile. Her blood pressure was 110/70 mmHg, and heart rate was 80 beats per minute. Physical examination revealed bilateral pedal edema. Deep tendon reflexes were equal and brisk bilaterally. Her chest was clear to auscultation. Pelvic examination was notable for a term-size mildly contracting uterus. The fetus was in vertex presentation, occiput posterior, with fetal heart sounds present. Urinalysis revealed no urine protein. During delivery, due to cephalopelvic disproportion, there was no fetal descent despite pushing for four hours, resulting in an arrested second stage of labor. As such, emergency lower segment cesarian section was preformed. The surgery went smoothly, without other complications noted.

One day after her delivery, the patient developed an erythematous maculopapular rash involving the chest and face, suggestive of urticaria. After administration of levocetirizine 5 mg at bedtime, the rash improved, completely disappearing three days after its emergence.

**Figure 1:**
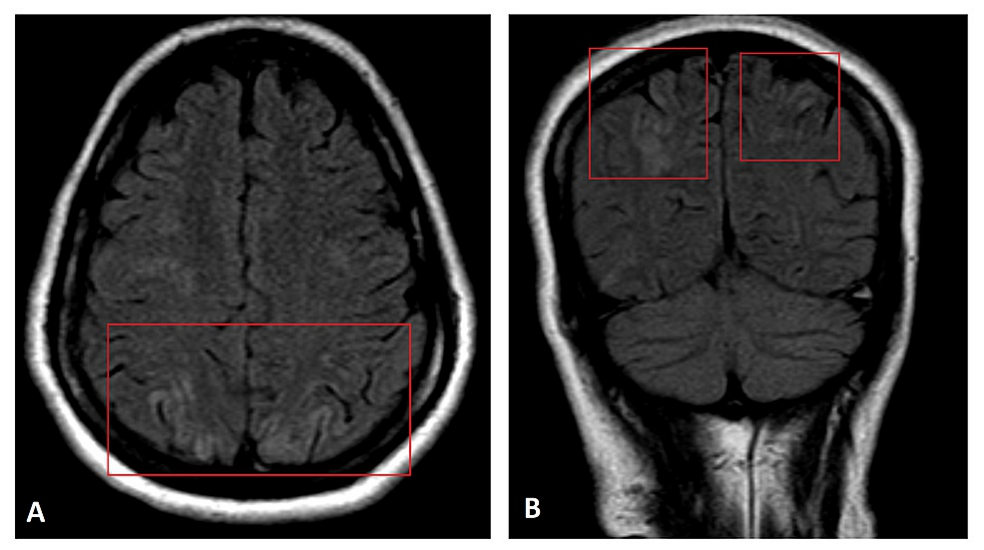
MRI FLAIR axial (A) and coronal (B) images, taken one hour after evaluation by the emergency team, shows multiple ill-defined scattered areas of FLAIR hyperintensities (red boxes) seen predominantly in the bilateral posterior parietal and occipital lobes MRI, magnetic resonance imaging; FLAIR, fluid-attenuated inversion recovery

**Figure 2:**
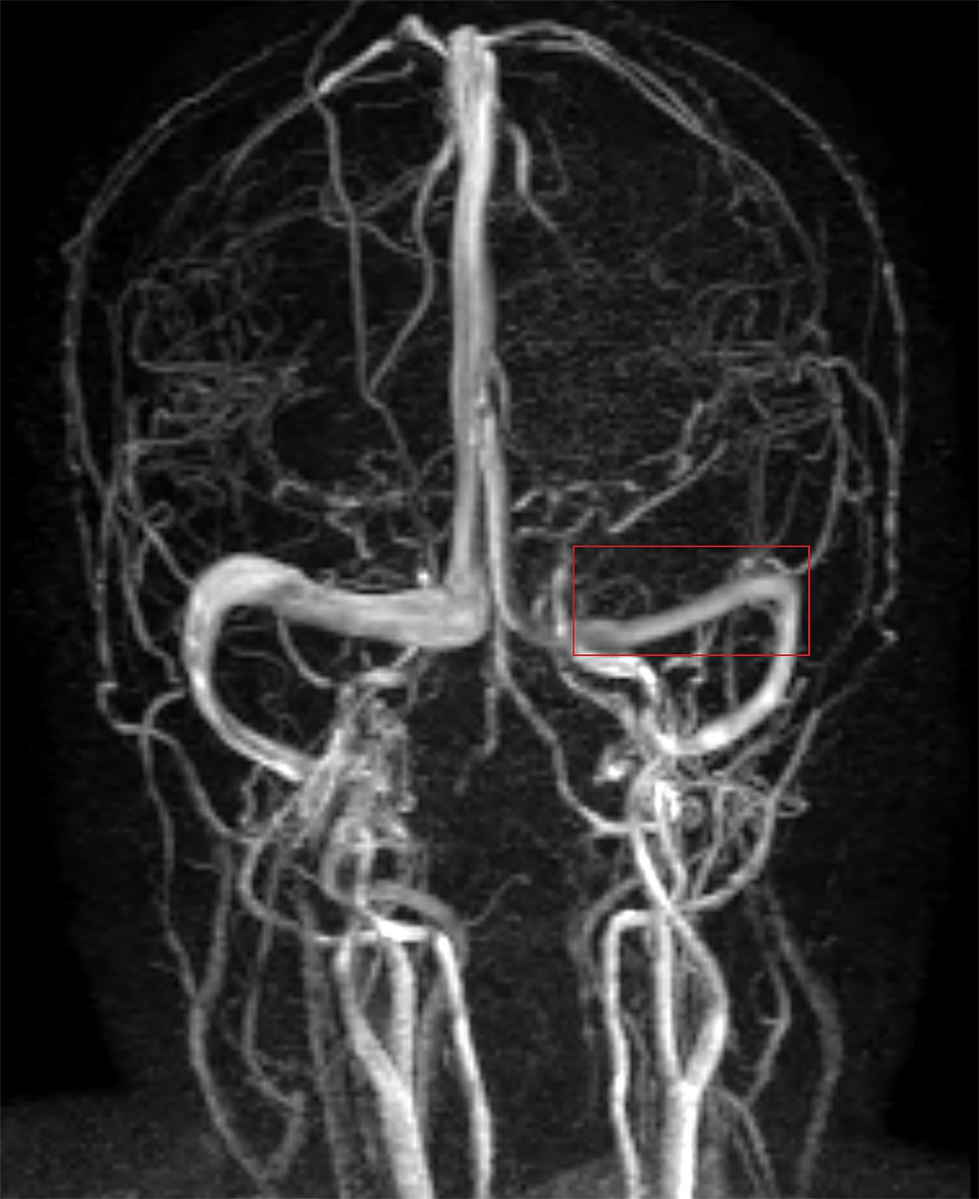
Magnetic resonance venogram, taken one hour after evaluation by the emergency team, revealed no abnormalities apart from hypoplastic left transverse sinuses (red box)

Five days after delivery, the patient developed a blood pressure of 140/90 mmHg, and was given labetalol 100 mg twice a day. At the time, she denied any headaches, visual disturbances or altered mental status. Deep tendon reflexes were equal and brisk bilaterally. The next morning, the patient developed a generalized tonic-clonic seizure. Upon evaluation by the medical emergency team, the patient was in a postictal state, and reported headache and transient visual disturbances. Her blood pressure was 120/80, heart rate 104 beats/minute and SpO_2_ 97% at the time. Rescue measures were instituted promptly, and included securing the oral airway and administering IV 25% dextrose 100 mL, IV midazolam 2 mg, and 4 g magnesium sulfate in IV 20% solution. Emergency laboratory testing revealed a lactate dehydrogenase level of 243 U/L, uric acid 3.9 mg/dL, 24-hour fluid intake of 2950 mL and 24-hour urine output of 2850 mL. Urinalysis revealed a urine protein to creatinine ratio of 3.81 g/gCr. Capillary blood glucose was low at 25 mg/dL. Red blood cell count, platelet count, hemoglobin levels, renal function, and coagulation panel were all within the normal range. Fundoscopy revealed no evidence of papilledema. MRI of the brain revealed multiple ill-defined scattered areas of fluid-attenuated inversion recovery (FLAIR) hyperintensities with no diffusion restriction involving predominantly the bilateral posterior parietal, occipital and frontal lobes, features suggestive of posterior reversible encephalopathy syndrome (Figure 1). Diffusion-weighted imaging of corresponding areas revealed no diffusion restriction, thus ruling out cerebral infarction. Magnetic resonance venography revealed no abnormalities apart from hypoplastic left transverse and sigmoid sinuses (Figure 2). The magnetic resonance angiogram revealed no abnormalities.

The patient was started on levetiracetam 500 mg twice a day for seven days. The patient’s condition improved markedly on the second day after her seizure, and she was discharged with levetiracetam 500 mg twice a day, calcium and ferrous sulfate. The final diagnosis of this patient was (1) late postpartum eclampsia and (2) posterior reversible encephalopathy syndrome.

## Discussion

Posterior reversible encephalopathy syndrome is a rare diagnosis that can develop in the postpartum period [7]. Patients tend to present with non-specific neurological signs such as headache and visual disturbances; it may lead to seizures or loss of consciousness [2]. Eclampsia and pre-eclampsia are one of the most important causes of posterior reversible encephalopathy syndrome in the obstetric population [5]. While pre-eclampsia and eclampsia typically arise during the second and third trimesters of pregnancy, they can also develop during the puerperium, like in our patient.

Posterior reversible encephalopathy syndrome is thought to be associated with an endothelial injury that contributes to abnormal placental development in patients with pre-eclampsia and eclampsia [9]. The dysregulation of microRNAs might lead to this abnormal placental development. MicroRNAs are small RNA molecules that are responsible for regulating biological pathways such as cell development and differentiation. An impaired regulation of microRNAs may lead to endothelial dysfunction, inflammation and abnormal placental vascularization [10]. Endothelial dysfunction may also be generalized and impact the cerebral tissues through several mechanisms: trans-endothelial leakage, localized exposure to free radicals, hypoxia, and cerebral edema [11]. Eclamptic seizures can occur if the motor cortex is affected, and posterior reversible encephalopathy syndrome may develop if the occipital cortex is involved [3].

Postpartum patients with a combination of headache, visual disturbances, edema and/or seizures should be evaluated for posterior reversible encephalopathy syndrome [9]. Our patient developed eclampsia and posterior reversible encephalopathy syndrome five days after her delivery. Prior to this event, she did not report any specific symptoms of pre-eclampsia such as visual disturbances, shortness of breath, headache, decreased urine output and altered mental status. She did not have any history of hypertension or pre-eclampsia during her pregnancy, and monitoring throughout the postpartum period revealed normal blood pressure until she was five days post partum. Our patient’s presentation highlights the unpredictability of eclampsia and posterior reversible encephalopathy syndrome [12].

Brain magnetic resonance imaging is the best imaging modality for posterior reversible encephalopathy syndrome; it can identify lesions associated with the condition [4]. Magnetic resonance venography and arteriography should also be conducted to exclude other disorders such as cerebral venous sinus thrombosis - the most frequent cerebrovascular disorder in the postpartum period [10]. Posterior reversible encephalopathy syndrome is typically managed with antihypertensive medication [1]. The medication provided is based on general recommendations for the management of hypertensive emergency, with a 24-hour goal blood pressure reduction of 25% [1]. Anticonvulsive medication is also required. There are no general recommendations for using a specific antiepileptic drug. Anticonvulsants can be tapered off as soon as the patient is asymptomatic, and lesions on MRI imaging have fully reversed [13]. Magnesium sulfate is also frequently administered for its anticonvulsive and vasodilating effects [13].

While the prognosis of posterior reversible encephalopathy syndrome is good, patients may develop neurological sequalae such as intracranial hemorrhage status epilepticus and cerebral ischemia [1]. As such, prompt identification and management of posterior reversible encephalopathy syndrome is necessary. Our patient benefitted from the administration of labetalol, levetiracetam and magnesium sulfate, having fully recovered one week after her eclamptic episode and . She reported no further neurological sequelae during her follow-up visit one month after discharge, and MRI performed three weeks after discharge revealed no abnormalities.

Early detection and prevention of posterior reversible encephalopathy syndrome is important, as it is associated with both maternal and fetal morbidity and mortality. Serum biomarkers such as endothelial progenitor cells, natural killer cells, neurokinin B, miRNAs, placental growth factor (PlGF), soluble endoglin and soluble fms-like tyrosine kinase 1 (sFlt-1) may be used to predict the presence of pre-eclampsia in women [14]. A sFlt-1/PlGF ratio may even be used to predict the short-term absence of pre-eclampsia in women [15].

Women at high risk of pre-eclampsia and posterior reversible encephalopathy syndrome may also benefit from preventative measures. According to the ASPRE (Combined Multimarker Screening and Randomized Patient Treatment with Aspirin for Evidence-Based Preeclampsia Prevention) trial, low-dose aspirin given to women who are at high risk of pre-eclampsia from 11 to 36 weeks may help prevent pre-eclampsia [16]. Our patient was taking aspirin throughout her pregnancy. However, it was discontinued shortly before delivery. It is not known if eclampsia and posterior reversible encephalopathy syndrome could have been prevented had she been on aspirin after delivery. Further studies to examine the utility and safety of aspirin prophylaxis during the postpartum period may be warranted.

## Conclusions

The case described here highlights the clinical presentation and management of posterior reversible encephalopathy syndrome in the obstetric postpartum population. Patients who present with neurological symptoms, such as seizures, disturbed vision and headache, in the postpartum period, should be evaluated for pre-eclampsia and posterior reversible encephalopathy syndrome. The mainstay management of posterior reversible encephalopathy syndrome includes magnesium sulfate, antihypertensive medication, and anticonvulsants. Aspirin may also be used to prevent the development of eclampsia and posterior reversible encephalopathy syndrome. Further research is needed to better understand the pathophysiology and prevention of eclampsia and posterior reversible encephalopathy syndrome.
